# Performance analysis of electronic power transformer based on neuro-fuzzy controller

**DOI:** 10.1186/s40064-016-2972-0

**Published:** 2016-08-15

**Authors:** Hakan Acikgoz, O. Fatih Kececioglu, Ceyhun Yildiz, Ahmet Gani, Mustafa Sekkeli

**Affiliations:** 1 Department of Electrical Science, Kilis 7 Aralik University, Kilis, 79000 Turkey; 2 Department of Electrical and Electronics, Faculty of Engineering, Kahramanmaras Sutcu Imam University, Kahramanmaras, Turkey

**Keywords:** Transformers, Power electronic transformer, Neuro-fuzzy controller, PWM rectifier, DAB converter

## Abstract

In recent years, electronic power transformer (EPT), which is also called solid state transformer, has attracted great interest and has been used in place of the conventional power transformers. These transformers have many important functions as high unity power factor, low harmonic distortion, constant DC bus voltage, regulated output voltage and compensation capability. In this study, proposed EPT structure contains a three-phase pulse width modulation rectifier that converts 800 V_rms_ AC to 2000 V DC bus at input stage, a dual active bridge converter that provides 400 V DC bus with 5:1 high frequency transformer at isolation stage and a three-phase two level inverter that is used to obtain AC output at output stage. In order to enhance dynamic performance of EPT structure, neuro fuzzy controllers which have durable and nonlinear nature are used in input and isolation stages instead of PI controllers. The main aim of EPT structure with the proposed controller is to improve the stability of power system and to provide faster response against disturbances. Moreover, a number of simulation results are carried out to verify EPT structure designed in MATLAB/Simulink environment and to analyze compensation ability for voltage harmonics, voltage flicker and voltage sag/swell conditions.

## Background

Generation, transmission and distribution of electrical energy are the most important factors in modern energy systems and transformers provide the most important role in these systems. Transformers which carry out many fundamental tasks such as galvanic isolation, voltage transformation, noise decoupling are widely used in electric power systems. It is well-known that classic (50/60 Hz) transformers have many positive features such as high efficiency, low cost and high reliability (Ronan et al. 2002; Yang et al. 2015; Zhao et al. 2013; Wang et al. 2007; Hwang et al. 2013). However, these conventional transformers have many undesirable drawbacks. These drawbacks include: (1) Conventional transformers have large size and weight because of their copper windings and iron core. (2) Conventional transformers are a passive component between the high and low voltages. Therefore, when voltage sags and swells occur at the input side, the output side is affected by these conditions. Harmonics in the output currents affect the input currents of transformers. In this case, harmonics can spread to the grid or can increase losses in the primary winding. Therefore, transformers have poor voltage regulation and low harmonic isolation. (3) Mineral oils used in transformers can be harmful when exposed to the environment in case of any fault in the transformer.

In recent years, with rapid advances in microprocessors and power electronics devices, many studies have been realized in order to improve performance of transformers. A new transformer was proposed by McMurray (1970). These transformers were called as electronic power transformer (EPT) or solid state transformer (SST). The main feature of these transformers is having ability to perform the same tasks with conventional transformers. Besides, EPTs possess many advantages over conventional transformers such as voltage sag and swell compensations, fixed AC output voltage, instantaneous voltage regulation, power factor correction, reactive power compensation, harmonic isolation and all of these advantages can be realized on a single circuit (Bifaretti et al. 2011; Dujic et al. 2013; Kang et al. 1999; Kececioglu et al. 2016; Grider et al. 2011; Xu et al. 2014; Acikgoz and Sekkeli 2014; Zhao et al. 2014; Yang et al. 2015). Many studies which focus on design and control of EPT structures have been realized by many researchers and institutes in the literature. In generally, two approaches were proposed for EPT structure; with DC-link and without DC-link (Falcones et al. 2010). EPT structure with DC-link consisting input, isolation and output stages has several key features such as reactive power and voltage sag/swell compensations (Yang et al. 2015; Lai et al. 2005). Pulse width modulation (PWM) rectifiers are widely used at input stage of these EPT structures to convert AC voltage into DC voltage because of their good dynamic response, unity power factor and regulated DC bus voltage. Isolation stage has DC–DC converter and high frequency (HF) transformer, and output stage has single or three-phase inverter which generates the desired output voltage and power (Falcones et al. 2010; Yang et al. 2015; Hwang et al. 2013).

During the last decades, intelligent control systems have been used in various applications. Neuro and fuzzy controllers have been outstanding intelligent control systems. Complexity and uncertainty of systems have promoted researchers to develop intelligent and adaptive control systems. Within this scope, many studies have been carried out to analyze performances of intelligent control systems (Jang et al. 1997). These control systems have been applied to many control systems and it has been obtained successful results. Development of intelligent control systems has milestones. Zadeh (1965) proposed fuzzy sets concept. This concept has led up the development of control systems that have of human reasoning capability. McCulloch and Pitts (1943) developed mimic biological neural systems computational abilities. Control systems have gained learning capability by this technique. Another approach is neuro-fuzzy controller (NFC). NFC that has nonlinear, robust structure and based on FLC whose functions are realized by ANN is one of these intelligent controllers (Jang et al. 1997; Mohagheghi et al. 2007; Tuncer and Dandil 2008). The most important feature of this controller is that it does not need the mathematical model of the controlled system.

In control of PWM rectifiers, DC bus voltage and dq-axis currents are commonly controlled by using Proportional-Integral (PI) controller because of its simple structure (Dannehl et al. 2009; Blasko and Kaura 1997). However, PI controller has undesirable features including slow response, large overshoots, oscillations, and it needs a mathematical model of the system to be controlled. Recently, intelligent and robust controllers, based on fuzzy logic controller (FLC), linear quadratic regulator (LQR), sliding mode controller (SMC), Robust H∞ controller and predictive control (PC), have been successfully used in many studies. To obtain a good performance from EPT structure, intelligent controllers can be used in transient and steady-state conditions (Bouafia and Krim 2008; Bouafia et al. 2010; Yu et al. 2010; Brando et al. 2010; Zhao et al. 2012; Djerioui et al. 2014; Liu et al. 2009; Hooshmand et al. 2012).

In this paper, robust and nonlinear control strategy based NFC controller is proposed for EPT structure in order to achieve a good dynamic response. Designed NFCs have two inputs, single output and six layers. This paper is organized as follows. Power circuit and mathematical model of EPT structure is given in section two. The description of the NFC and its training algorithm are explained in section three. The simulation results related to the proposed EPT structure are comprehensively presented in section four. Section five provides the conclusions of this study.

## Mathematical model of EPT

**Fig. 1:**
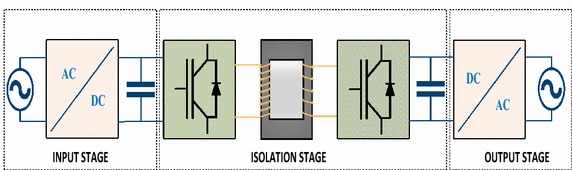
Configuration of proposed EPT structure

In this section, block diagram of proposed EPT structure is consisted of input, isolation and output stages and has AC/DC/AC/DC/AC conversions as seen Fig. 1.

**Fig. 2:**
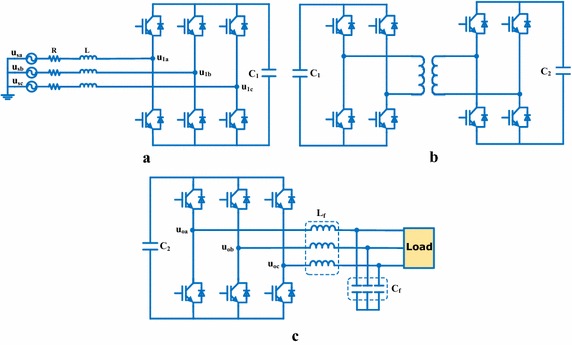
Sections of EPT structure. **a** Input stage, **b** isolation stage and **c** output stage

Input stage is the most important part of EPT structures and three-phase AC voltage is rectified by PWM rectifier at this stage (Liu et al. 2009; Hooshmand et al. 2012). There are many control methods for PWM rectifier in the literature. Voltage source PWM rectifier is preferred in this study in order to control DC bus voltage in input stage. PI controllers are often used in PWM rectifier due to their simple structures (Singh et al. 2004; Blasko and Kaura 1997). In this paper, NFC that has a more durable construction is designed instead of PI controller. So, EPT structure will be more durable and will be provide faster response against all disturbances. DC voltage rectified input stage is converted into high frequency square wave by using DC–DC converter. DC voltage obtained from isolation stage is transmitted to two-level inverter. The inverter provides the power and voltage required for the load. Moreover, stages of EPT structure are shown in detail in Fig. 2.

The voltage equations of EPT structure are expressed as following:

1 $$\left[ {\begin{array}{*{20}c} {{\text{u}}_{\text{sa}} (\rm t)} \\ {{\text{u}}_{\text{sb}} (\rm t)} \\ {{\text{u}}_{\text{sc}} (\rm t)} \\ \end{array} } \right] = \sqrt 2 U_{s} \, \left[ {\begin{array}{*{20}c} {\sin \omega \rm t} \\ {\sin (\omega \rm t - 120)} \\ {\sin (\omega \rm t + 120)} \\ \end{array} } \right]$$

2 $$\left[ {\begin{array}{*{20}c} {{\text{u}}_{\text{la}} ({\text{t}})} \\ {{\text{u}}_{\text{lb}} ({\text{t}})} \\ {{\text{u}}_{\text{lc}} ({\text{t}})} \\ \end{array} } \right] = {\text{m}}_{1} {\text{U}}_{\text{dc}} \, \left[ {\begin{array}{*{20}c} {{\text{sin (}}\omega {\text{t}} - \theta_{ 1} )} \\ {{ \sin }(\omega {\text{t}} - 120 - \theta_{ 1} )} \\ {{ \sin }(\omega {\text{t}} + 120 - \theta_{ 1} )} \\ \end{array} } \right]$$

3 $$\left[ {\begin{array}{*{20}c} {{\text{u}}_{\text{oa}} ({\text{t}})} \\ {{\text{u}}_{\text{ob}} ({\text{t}})} \\ {{\text{u}}_{\text{oc}} ({\text{t}})} \\ \end{array} } \right] = {\text{m}}_{2} {\text{U}}_{\text{dc}} \,\,\left[ {\begin{array}{*{20}c} {{\text{sin (}}\omega {\text{t}} - \theta_{ 2} )} \\ {{ \sin }(\omega {\text{t}} - 120 - \theta_{ 2} )} \\ {{ \sin }(\omega {\text{t}} + 120 - \theta_{ 2} )} \\ \end{array} } \right]$$

According to Fig. 1 and transformation ratio of HF transformer used in isolation stage, the dynamic differential equations of EPT structure can be achieved in matrix forms as:

4 $${\text{L}}\frac{\text{d}}{\text{dt}} \, \left[ {\begin{array}{*{20}c} {{\text{i}}_{\text{la}} ({\text{t}})} \\ {{\text{i}}_{\text{lb}} ({\text{t}})} \\ {{\text{i}}_{\text{lc}} ({\text{t}})} \\ \end{array} } \right] = \, \left[ {\begin{array}{*{20}c} {{\text{u}}_{\text{sa}} ({\text{t}})} \\ {{\text{u}}_{\text{sb}} ({\text{t}})} \\ {{\text{u}}_{\text{sc}} ({\text{t}})} \\ \end{array} } \right] - \, \left[ {\begin{array}{*{20}c} {{\text{u}}_{\text{la}} ({\text{t}})} \\ {{\text{u}}_{\text{lb}} ({\text{t}})} \\ {{\text{u}}_{\text{lc}} ({\text{t}})} \\ \end{array} } \right] - {\text{R }}\left[ {\begin{array}{*{20}c} {{\text{i}}_{\text{la}} ({\text{t}})} \\ {{\text{i}}_{\text{lb}} ({\text{t}})} \\ {{\text{i}}_{\text{lc}} ({\text{t}})} \\ \end{array} } \right]$$

5 $${\text{L}}_{\text{f}} \frac{\text{d}}{\text{dt}} \, \left[ {\begin{array}{*{20}c} {{\text{i}}_{\text{fa}} ( {\text{t)}}} \\ {{\text{i}}_{\text{fb}} ( {\text{t)}}} \\ {{\text{i}}_{\text{fc}} ( {\text{t)}}} \\ \end{array} } \right] = \frac{ 1}{\text{k}} \, \left[ {\begin{array}{*{20}c} {{\text{u}}_{\text{oa}} ( {\text{t)}}} \\ {{\text{u}}_{\text{ob}} ( {\text{t)}}} \\ {{\text{u}}_{\text{oc}} ( {\text{t)}}} \\ \end{array} } \right] - \, \left[ {\begin{array}{*{20}c} {{\text{u}}_{\text{La}} ( {\text{t)}}} \\ {{\text{u}}_{\text{Lb}} ( {\text{t)}}} \\ {{\text{u}}_{\text{Lc}} ( {\text{t)}}} \\ \end{array} } \right]$$

6 $${\text{C}}_{\text{f}} \frac{\text{d}}{\text{dt}} \, \left[ {\begin{array}{*{20}c} {{\text{u}}_{\text{La}} ( {\text{t)}}} \\ {{\text{u}}_{\text{Lb}} ( {\text{t)}}} \\ {{\text{u}}_{\text{Lc}} ( {\text{t)}}} \\ \end{array} } \right] = \, \left[ {\begin{array}{*{20}c} {{\text{i}}_{\text{fa}} ( {\text{t)}}} \\ {{\text{i}}_{\text{fb}} ( {\text{t)}}} \\ {{\text{i}}_{\text{fc}} ( {\text{t)}}} \\ \end{array} } \right] - \, \left[ {\begin{array}{*{20}c} {{\text{i}}_{\text{La}} ( {\text{t)}}} \\ {{\text{i}}_{\text{Lb}} ( {\text{t)}}} \\ {{\text{i}}_{\text{Lc}} ( {\text{t)}}} \\ \end{array} } \right]$$

7 $$\frac{\text{d}}{\text{dt}} \, \left( {\frac{1}{2}{\text{C}}_{\text{dc}}^{2} ({\text{t}})} \right) = \left[ {\begin{array}{*{20}c} {{\text{u}}_{\text{la}} ({\text{t}})} & {{\text{u}}_{\text{lb}} ({\text{t}})} & {{\text{u}}_{\text{lc}} ({\text{t}})} \\ \end{array} } \right] \, \left[ {\begin{array}{*{20}c} {{\text{i}}_{\text{la}} ({\text{t}})} \\ {{\text{i}}_{\text{lb}} ({\text{t}})} \\ {{\text{i}}_{\text{lc}} ({\text{t}})} \\ \end{array} } \right] - \frac{1}{\text{k}} \, \left[ {\begin{array}{*{20}c} {{\text{u}}_{\text{oa}} ({\text{t}})} & {{\text{u}}_{\text{ob}} ({\text{t}})} & {{\text{u}}_{\text{oc}} ({\text{t}})} \\ \end{array} } \right] \left[ {\begin{array}{*{20}c} {{\text{i}}_{\text{fa}} ({\text{t}})} \\ {{\text{i}}_{\text{fb}} ({\text{t}})} \\ {{\text{i}}_{\text{fc}} ({\text{t}})} \\ \end{array} } \right]$$

Where, k is transformation ratio of HF transformer. m_1_ and ϴ_1_ are amplitude modulation index and modulation angle for PWM rectifier used in the input stage. m_2_ and ϴ_2_ are amplitude modulation index and modulation angle for PWM inverter used in the output stage. u_sa_, u_sb_ and u_sc_ are input voltages. u_La_, u_Lb_ and u_Lc_ are output voltages. U_dc_ is DC bus voltage of input stage. u_0a_, u_0b_ and u_0c_ are AC voltages in the output stage. u_la_, u_lb_ and u_lc_ are AC voltages in the input stage (Liu et al. 2009; Hooshmand et al. 2012; Acikgoz et al. 2015). According to three-phase stationary reference frame a-b-c, dynamic model of proposed EPT structure can obtained by Eqs. (1) to (7). However, the parameters of the dynamic differential equations are time-varying and should be transformed to the synchronously rotating reference frame using Park’s transformer in order to obtain time-invariant equations (Liu et al. 2009; Hooshmand et al. 2012). Thus, the dynamic equations in the d-q rotating reference frame are as follows:

8 $$\frac{\text{d}}{\text{dt}}\left[ {\begin{array}{*{20}c} {{\text{i}}_{\text{ld}} ({\text{t}})} \\ {{\text{i}}_{\text{lq}} ({\text{t}})} \\ \end{array} } \right] = - \frac{\text{R}}{\text{L}} \, \left[ {\begin{array}{*{20}c} {{\text{i}}_{\text{ld}} } \\ {{\text{i}}_{\text{lq}} } \\ \end{array} } \right] - \omega \;\left[ {\begin{array}{*{20}c} {{\text{i}}_{\text{lq}} } \\ { - {\text{i}}_{\text{ld}} } \\ \end{array} } \right] + \frac{{{\text{m}}_{1} }}{\text{L}}{\text{u}}_{\text{dc}} \;\left[ {\begin{array}{*{20}c} {\sin \;\theta_{1} } \\ {\cos \;\theta_{1} } \\ \end{array} } \right] + \frac{\sqrt 2 }{\text{L}} \, \left[ {\begin{array}{*{20}c} 0 \\ {{\text{u}}_{\text{s}} } \\ \end{array} } \right]$$

9 $$\frac{\text{d}}{\text{dt}} \, \left[ {{\text{u}}_{\text{dc}} } \right] = - \frac{{3{\text{m}}_{1} }}{{2{\text{C}}}} \, \left[ {\begin{array}{*{20}c} {{\text{i}}_{\text{ld}} \;\sin \;\theta_{1} } \\ { - {\text{i}}_{\text{lq}} \;\cos \;\theta_{1} } \\ \end{array} } \right] + \frac{{3{\text{m}}_{2} }}{{2{\text{kC}}}} \, \left[ {\begin{array}{*{20}c} {{\text{i}}_{\text{fd}} \;\sin \;\theta_{2} } \\ { - {\text{i}}_{\text{fq}} \;\cos \;\theta_{2} } \\ \end{array} } \right]$$

10 $$\frac{\text{d}}{\text{dt}}\left[ {\begin{array}{*{20}c} {{\text{u}}_{\text{Ld}} } \\ {{\text{u}}_{\text{Lq}} } \\ \end{array} } \right] = \frac{1}{{{\text{C}}_{\text{f}} }} \, \left[ {\begin{array}{*{20}c} {{\text{i}}_{\text{fd}} } \\ {{\text{i}}_{\text{fq}} } \\ \end{array} } \right] - \frac{1}{{{\text{C}}_{\text{f}} }} \, \left[ {\begin{array}{*{20}c} {{\text{i}}_{\text{Ld}} } \\ {{\text{i}}_{\text{Lq}} } \\ \end{array} } \right] - \omega \left[ {\begin{array}{*{20}c} {{\text{u}}_{\text{Lq}} } \\ { - {\text{u}}_{\text{Ld}} } \\ \end{array} } \right]$$

11 $$\frac{\text{d}}{\text{dt}}\left[ {\begin{array}{*{20}c} {{\text{i}}_{\text{fd}} } \\ {{\text{i}}_{\text{fq}} } \\ \end{array} } \right] = \omega \left[ {\begin{array}{*{20}c} { - {\text{i}}_{\text{fq}} } \\ {{\text{i}}_{\text{fd}} } \\ \end{array} } \right] + \frac{{{\text{m}}_{2} \sin \theta_{2} }}{{{\text{kL}}_{\text{f}} }} \, \left[ {\begin{array}{*{20}c} { - {\text{u}}_{\text{dc}} } \\ {{\text{u}}_{\text{dc}} } \\ \end{array} } \right] - \frac{1}{{{\text{L}}_{\text{f}} }} \, \left[ {\begin{array}{*{20}c} {{\text{u}}_{\text{Ld}} } \\ {{\text{u}}_{\text{Lq}} } \\ \end{array} } \right]$$

where, $$\left[ {{\text{i}}_{\text{ld }} {\text{i}}_{\text{lq}} {\text{i}}_{\text{lo}} } \right]^{\text{T}} = {\text{K }}\left[ {{\text{i}}_{\text{la }} {\text{i}}_{\text{lb}} {\text{i}}_{\text{lc}} } \right]^{\text{T}} ,$$$$\left[ {{\text{i}}_{\text{fd }} {\text{i}}_{\text{fq}} {\text{i}}_{\text{fo}} } \right]^{\text{T}} = {\text{K }}\left[ {{\text{i}}_{\text{fa }} {\text{i}}_{\text{fb}} {\text{i}}_{\text{fc}} } \right]^{\text{T}} ,\left[ {{\text{i}}_{\text{Ld }} {\text{i}}_{\text{Lq}} {\text{i}}_{\text{Lo}} } \right]^{\text{T}} = {\text{K }}\left[ {{\text{i}}_{\text{La }} {\text{i}}_{\text{Lb}} {\text{i}}_{\text{Lc}} } \right]^{\text{T}}$$$$\left[ {{\text{u}}_{\text{Ld }} {\text{u}}_{\text{Lq}} {\text{u}}_{\text{Lo}} } \right]^{\text{T}} = {\text{K }}\left[ {{\text{u}}_{\text{La }} {\text{u}}_{\text{Lb}} {\text{u}}_{\text{Lc}} } \right]^{\text{T}}$$K is the Park’s transformation matrix given by:

12 $${\text{K}} = \frac{2}{3}\left[ {\begin{array}{*{20}c} {\cos \,\omega {\text{t}}} & \quad{\cos \,(\omega {\text{t}} - 120)} & \quad{\cos \,(\omega {\text{t}} + 120)} \\ {\sin \,\omega {\text{t}}} & \quad{\sin \,(\omega {\text{t}} - 120)} & \quad{\sin \,(\omega {\text{t}} + 120)} \\ {\frac{3}{2}} & \quad{\frac{3}{2}} & \quad{\frac{3}{2}} \\ \end{array} } \right]$$

## Implementation and design of neuro-fuzzy controller

NFCs based on the principle that the functions of fuzzy logic controller (FLC) are performed artificial neural network (ANN) are successfully applied to many industrial applications (Zadeh 1965; Jang et al. 1997; Mohagheghi et al. 2007; Tuncer and Dandil 2008). In addition, NFCs have a non-linear structure and do not need mathematical model of the system to be controlled. Thus, NFCs are commonly used in non-linear systems with parameter variation and uncertainty. Fuzzy rules of sugeno type fuzzy logic are defined as below:

13 $${\text{R}}^{\text{j}} :{\text{If X}}_{ 1} ,{\text{A}}_{1}^{\text{j}} , \ldots {\text{X}}_{\text{n}} ,{\text{A}}_{\text{n}}^{\text{j}} \quad {\text{then}}\;{\text{ y}} = {\text{f}}_{\text{i}} = a_{0}^{\text{j}} + a_{1}^{\text{j}} {\text{X}}_{ 1} + a_{2}^{\text{j}} {\text{X}}_{ 2} + a_{\text{n}}^{\text{j}} {\text{X}}_{\text{n}}$$

**Fig. 3:**
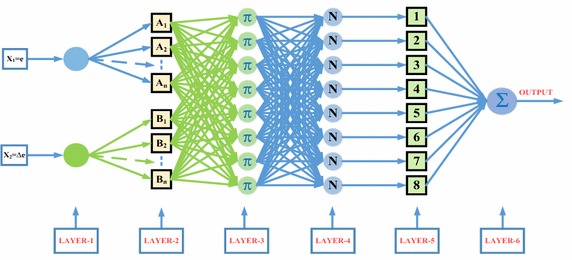
Two-input Sugeno type NFC structure

Here, X_i_ is the input variable, y is the output variable, linguistic variables of prerequisites with A_i_^j^ µ_Ai_^j^ (x_i_) membership function and the A_i_^j^ ϵ R are the coefficients of linear f_i_ = (x_1_, x_2_, …, x_n_) function. Structure of NFC which is used in control algorithms is shown in Fig. 3. As seen in Fig. 3, NFC has two inputs, one output and six layers. Five membership functions were chosen for each input (Jang et al. 1997; Tuncer and Dandil 2008; Buckley and Hayashi 1994).

Membership functions are performed in the second layer where membership function is replaced by activation function of each artificial neuro cell. Five membership functions are determined for the error and the change of error. The output of this layer is obtained as follows:

14 $${\text{net}}_{\text{j}}^{2} = - \frac{{({\text{x}}_{\text{i}} - {\text{m}}_{\text{ij}} )^{2} }}{{2(\sigma_{\text{ij}} )^{2} }},\quad y_{\text{j}}^{2} = \exp \, ({\text{net}}_{\text{j}}^{2} )$$

σ_ij_ and m_ij_, which are input parameters, represent the parameters of membership functions to be adapted. X_i_ represents the input of ith cell of second layer. Similar to FLC, the third layer of NFC consists of rule base and fuzzy rules are determined in this layer.

15 $${\text{net}}_{\text{k}}^{3} = \mathop \Pi \limits_{\text{j}} {\text{w}}_{\text{jk}}^{3} {\text{x}}_{\text{j}}^{3} ,\quad {\text{y}}_{\text{k}}^{3} = {\text{net}}_{\text{k}}^{3}$$

X_j_^3^ here represents the input of jth cell of the third layer. The output of the system defined by using central clarification for Mamdani fuzzy logic:

16 $${\text{net}}_{0}^{4} = \mathop \Sigma \limits_{\text{k}} {\text{w}}_{\text{k0}}^{4} {\text{y}}_{\text{k}}^{3} ,\quad {\text{y}}_{0}^{4} = \frac{{{\text{net}}_{0}^{4} }}{{\mathop \Sigma \limits_{\text{k}} {\text{y}}_{\text{k}}^{3} }}$$

Fourth layer is called normalization layer where the accuracy of fuzzy rules are calculated. Fifth layer is called firing size of a rule. The firing degree of normalized rules is multiplied by linear f function in this layer. This layer generates output values required for EPT structure. In order to update input and output parameters by using analog teaching method with back propagation algorithm, the squared error (E) which minimizes tracking error (e) is determined as follows (Jang et al. 1997):

17 $${\text{E}} = \frac{1}{2}{\text{e}}^{2}$$

The performance index for the parameters of membership functions in EPT structure can be derived as follows:

18 $$\frac{{\partial {\text{E}}}}{{\partial {\text{w}}_{{{\text{k}}0}} }} = - {\text{e}}.\text{sgn} \left( {\frac{{\Delta {\text{i}}_{\text{dq}} ,v_{\text{d}} ,\upvarphi }}{{\Delta {\text{y}}_{0}^{4} }}} \right)\frac{1}{{\mathop \Sigma \limits_{\text{k}} {\text{y}}_{\text{k}}^{3} }}{\text{w}}_{{{\text{k}}0}}^{4} \frac{{{\text{x}}_{\text{i}} - {\text{m}}_{\text{ij}} }}{{(\sigma_{\text{ij}} )^{2} }}{\text{y}}_{\text{j}}^{2}$$

19 $$\frac{{\partial {\text{E}}}}{{\partial \sigma_{\text{ij}} }} = - {\text{e}}.\text{sgn} \left( {\frac{{\Delta {\text{i}}_{\text{dq}} ,v_{\text{d}} ,\upvarphi }}{{\Delta {\text{y}}_{0}^{4} }}} \right)\frac{1}{{\mathop \Sigma \limits_{\text{k}} {\text{y}}_{\text{k}}^{3} }}{\text{w}}_{{{\text{k}}0}}^{4} \frac{{({\text{x}}_{\text{i}} - {\text{m}}_{\text{ij}} )^{2} }}{{(\sigma_{\text{ij}} )^{3} }}{\text{y}}_{\text{j}}^{2}$$

As shown in Fig. 3, inputs of NFC were selected as the error and the change of error. Five membership functions are used for each input. In the proposed NFC structure, precondition parameters of membership layer have been trained in the simulation model. During the simulation studies, output parameters have been trained using back-propagation learning algorithm. These parameters are adapted until the desired performance is reached.

### Control of the input stage

**Fig. 4:**
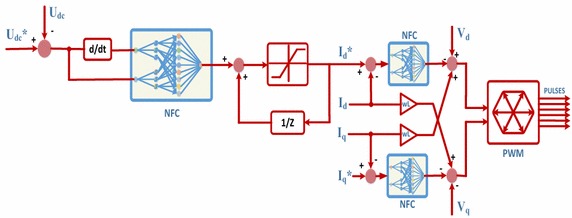
Control scheme of three-phase PWM rectifier

Three-phase PWM rectifier has been used in numerous applications in recent years. These rectifiers have many advantages such as bi-directional power flow, low harmonic distortion, unity power factor and control of DC-link voltage (Blasko and Kaura 1997; Dannehl et al. 2009). When considering all these features, three-phase PWM rectifier is the most important part in EPT structures. Control scheme of three-phase PWM rectifier based on NFC is as shown in Fig. 4. In control of DC voltage, DC bus voltage is compared with reference DC bus voltage. Error of DC bus voltages is applied to NFC controller. Reference value of d-axis current is obtained from output of NFC controller. In order to obtain unity power factor, reference value of q-axis current is set to zero. Error of dq-axis currents and changes of these errors are applied as input to NFCs. Afterwards, V_q_ and V_d_ values are obtained from the outputs of NFCs. These voltages are sent to PWM block, which generates required signals for driving the semiconductor-switching element. Moreover, an anti-wind up integrator is used to limit the output of NFC and compensate for steady state error (Liu et al. 2009; Hooshmand et al. 2012).

For PI controller, mathematical model of the input stage in the d–q rotating reference frame can be used as follows:

20 $${\text{L}}\frac{{{\text{d}}_{\text{ld}} }}{\text{dt}} = \omega {\text{LI}}_{\text{lq}} - {\text{u}}_{\text{ld}} + {\text{u}}_{\text{sd}} \,$$

21 $${\text{L}}\frac{{{\text{d}}_{\text{lq}} }}{\text{dt}} = - \omega {\text{LI}}_{\text{ld}} - {\text{u}}_{\text{lq}} + {\text{u}}_{\text{sq}} \,$$

where, $$\left[ {\begin{array}{*{20}c} {{\text{i}}_{\text{d}} } & {{\text{i}}_{\text{q}} } \\ \end{array} } \right]^{\text{T}} = {\text{K}}\left[ {\begin{array}{*{20}c} {{\text{i}}_{\text{la}} } & {{\text{i}}_{\text{lb}} } & {{\text{i}}_{\text{lc}} } \\ \end{array} } \right]^{\text{T}}$$, $$\left[ {\begin{array}{*{20}c} {{\text{u}}_{\text{ld}} } & {{\text{u}}_{\text{lq}} } \\ \end{array} } \right]^{\text{T}} = {\text{K}}\left[ {\begin{array}{*{20}c} {{\text{u}}_{\text{la}} } & {{\text{u}}_{\text{lb}} } & {{\text{u}}_{\text{lc}} } \\ \end{array} } \right]^{T}$$, $$\left[ {\begin{array}{*{20}c} {{\text{u}}_{\text{sd}} } & {{\text{u}}_{\text{sq}} } \\ \end{array} } \right]^{\text{T}} = {\text{K}}\left[ {\begin{array}{*{20}c} {{\text{u}}_{\text{sa}} } & {{\text{u}}_{\text{sb}} } & {{\text{u}}_{\text{sc}} } \\ \end{array} } \right]^{\text{T}}$$

22 $${\text{K}} = \frac{3}{2}\left[ {\begin{array}{*{20}c} {\sin \omega {\text{t}}} & {\sin (\omega {\text{t}} - 120)} & {\sin (\omega {\text{t}} + 120)} \\ {\cos \omega {\text{t}}} & {{ \cos }(\omega {\text{t}} - 120)} & {\cos (\omega {\text{t}} + 120)} \\ \end{array} } \right]$$

The grid voltage component of d-axis is equal to its peak value and q-axis of the grid voltage is equal to zero. Thus, d-axis of the current is equal to the active current component and q-axis of the current is equal to the reactive current component. Double loop control which has current and voltage loop is used in order to control of three-phase PWM rectifier. d-axis current is obtained from the DC voltage loop and q-axis current sets to zero in order to obtain power factor equal to “1”. For current loop controls, following equations can be written as:

23 $${\text{L}}\frac{{{\text{d}}_{\text{ld}} }}{\text{dt}} = \left( {{\text{K}}_{\text{p}} + \frac{{{\text{K}}_{\text{I}} }}{\text{s}}} \right)({\text{i}}_{\text{ld}}^{*} - {\text{i}}_{\text{ld}} )$$

24 $${\text{L}}\frac{{{\text{d}}_{\text{lq}} }}{\text{dt}} = \left( {{\text{K}}_{\text{p}} + \frac{{{\text{K}}_{\text{I}} }}{\text{s}}} \right){\text{i}}_{\text{lq}}$$

PI controller parameters of PWM rectifier used in the input stage are given in Table 1.

**Table 1:** Parameters of PI controllers

Parameters	Voltage control	Current control
Proportional gain (K_p_)	0.2	4
Integral gain (K_I_)	20	60

To analyze performance of three-phase PWM rectifier, a small signal model is used. First, the state variables are expressed as the sum of the values at an operating point and small deviations from the operating point (Ende and Shenghua 2013; Bel Hadj-Youssef et al. 2007):

25 $${\text{U}}_{\text{dc}} = {\text{U}}_{\text{dc}} + {\hat{\text{U}}}_{\text{dc}}$$

26 $${\text{U}}_{\text{d}} = {\text{U}}_{\text{d}} + {\hat{\text{U}}}_{\text{d}}$$

27 $${\text{i}}_{\text{d}} = {\text{i}}_{\text{d}} + {\hat{\text{i}}}_{\text{d}}$$

28 $${\text{i}}_{\text{L}} = {\text{i}}_{\text{L}} + {\hat{\text{i}}}_{\text{L}}$$

With the analysis of the mathematical model of the three-phase PWM rectifier, the model can be written in the form of Eqs. (29–30);

29 $${\text{L}}\frac{{{\text{di}}_{\text{d}} }}{\text{dt}} = \omega {\text{Li}}_{\text{q}} + {\text{e}}_{\text{d}} - {\text{u}}_{\text{d}}$$

30 $${\text{C}}\frac{{{\text{du}}_{\text{dc}} }}{\text{dt}} = \frac{3}{2}\frac{{({\text{u}}_{\text{d}} {\text{i}}_{\text{d}} + {\text{u}}_{\text{q}} {\text{i}}_{\text{q}} )}}{{{\text{u}}_{\text{dc}} }} - {\text{i}}_{\text{L}}$$

Using Eqs. (29) and (30), small signal model can be written as:

31 $$\left[ {\begin{array}{*{20}c} {{\hat{\text{u}}}_{\text{dc}} ({\text{s}})} \\ {{\text{i}}_{\text{d}} ({\text{s}})} \\ \end{array} } \right] = \left[ {\begin{array}{*{20}c} {\frac{{3{\text{i}}_{\text{d}} L - 3{\text{e}}_{\text{d}} }}{{2{\text{U}}_{\text{dc}} {\text{LCs}}^{2} }}} &\quad { - \frac{1}{\text{Cs}}} \\ { - \frac{1}{\text{L}}} & \quad0 \\ \end{array} } \right]\left[ {\begin{array}{*{20}c} {{\hat{\text{u}}}_{\text{d}} ({\text{s}})} \\ {{\hat{\text{i}}}_{\text{L}} ({\text{s}})} \\ \end{array} } \right]$$

**Fig. 5:**
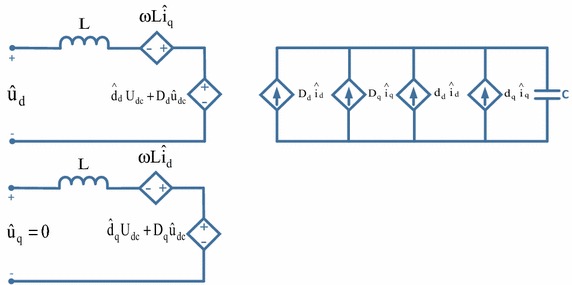
Small signal model of three-phase PWM rectifier

**Fig. 6:**
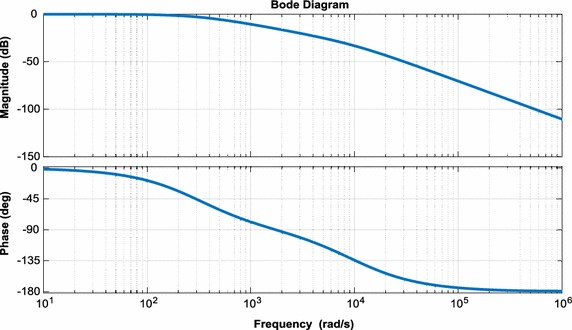
Bode plots of voltage loop for three-phase PWM rectifier

**Fig. 7:**
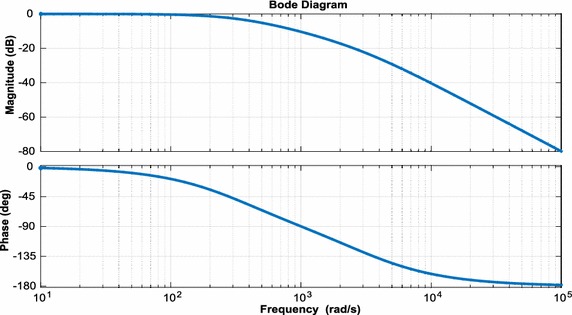
Bode plots of current loop for three-phase PWM rectifier

Small signal model of three-phase PWM rectifier has been derived and shown in Fig. 5. A diagram showing bode plots of the current and voltage loop for three-phase PWM rectifier are indicated in Figs. 6 and 7, which demonstrates that measured gain margins are infinite. Thus, these results show that the control system is stable.

### Control of the isolation stage

**Fig. 8:**
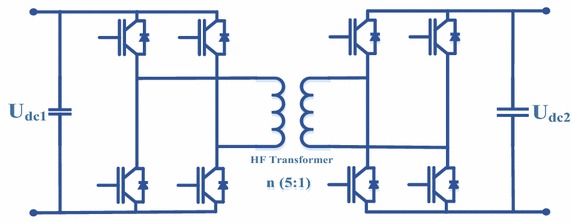
Configuration of DAB converter

**Fig. 9:**
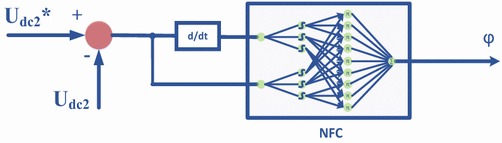
Phase shift control of DAB

DAB converter which provides high performance/efficiency, galvanic isolation and soft-switching property is used at the isolation stage to step down DC voltage obtained from input stage. The configuration of DAB converter is indicated in Fig. 8. DC voltage obtained from three-phase PWM rectifier is converted to a lower DC voltage using DAB converter. DC voltage obtained from the rectifier is first converted to the high-frequency square wave at isolation stage. According to the transformation ratio of HF transformer, this square wave is obtained in the same way at the secondary part of HF transformer. Then, this wave is converted into lower DC voltage by using DAB converter (Yang et al. 2015; Zhao et al. 2013). HF transformer provides electrical isolation and voltage transformation. In order to regulate DC voltage obtained from output of DAB converter, NFC and PI controller are used as phase shift controllers. The difference between DC voltage at the output of the isolation stage and reference DC voltage is compared and NFC/PI controller generates phase shift angle required for DAB converter as shown Fig. 9.

Moreover, the coefficients of PI controller used in isolation stage are given in Table 2.

**Table 2:** Parameters of PI controller for DAB

Parameters	Value
Proportional gain (K_p_)	0.002
Integral gain (K_I_)	6

**Fig. 10:**
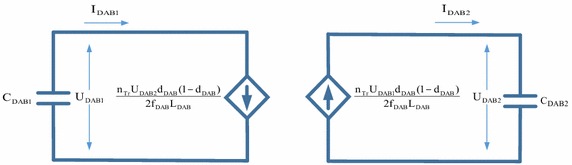
Small signal model of DAB converter

The equations of power, input and output currents required for DAB converter are as follows:

32 $${\text{P}}_{\text{DAB}} = \frac{{{\text{n}}_{\text{Tr}} {\text{U}}_{{{\text{DAB}}_{1} }} {\text{U}}_{{{\text{DAB}}2}} }}{{2{\text{f}}_{\text{DAB}} {\text{L}}_{\text{DAB}} }}{\text{d}}_{\text{DAB}} (1 - {\text{d}}_{\text{DAB}} )$$

33 $${\text{I}}_{{{\text{DAB}}1}} = \frac{{{\text{n}}_{\text{Tr}} {\text{U}}_{{{\text{DAB}}2}} }}{{2{\text{f}}_{\text{DAB}} {\text{L}}_{\text{DAB}} }}{\text{d}}_{\text{DAB}} (1 - {\text{d}}_{\text{DAB}} )$$

34 $${\text{I}}_{{{\text{DAB}}2}} = \frac{{{\text{n}}_{\text{Tr}} {\text{U}}_{{{\text{DAB}}1}} }}{{2{\text{f}}_{\text{DAB}} {\text{L}}_{\text{DAB}} }}{\text{d}}_{\text{DAB}} (1 - {\text{d}}_{\text{DAB}} )$$

where, U_DAB1_ and U_DAB2_ are input and output DC voltages of DAB converter, f_DAB_ is switching frequency of DAB converter, L_DAB_ is leakage inductance, d_DAB_ is ratio of time delay between two bridges to one-half of switching period (Yang et al. 2015; Zhao et al. 2013). Figure 10 shows small signal model of DAB converter.

**Fig. 11:**
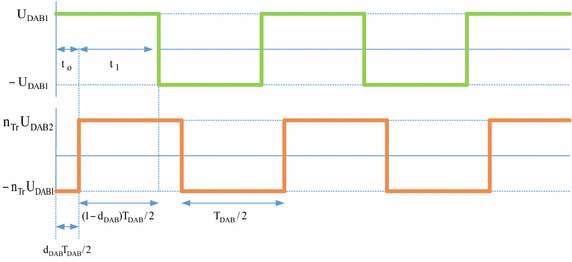
DAB converter waveforms

Small-signal model and transfer functions can be obtained by state-space averaging. A state-space averaging of DAB converter is described as linear combination of independent inputs and the physical state of its energy storage elements. Also, waveforms of DAB converter are shown in Fig. 11.

For small-signal model of DAB converter used in isolation stage of EPT structure, state-space equations are as follows:

35 $$\frac{\text{d}}{\text{dt}}{\text{x}} = {\text{A}}_{1} {\text{x}} + {\text{B}}_{1} {\text{u}} \Leftrightarrow \frac{\text{d}}{\text{dt}}\left[ {\begin{array}{*{20}c} {{\text{I}}_{{{\text{DAB}}2}} } \\ {{\text{U}}_{{{\text{DAB}}2}} } \\ \end{array} } \right] = \left[ {\begin{array}{*{20}c} { - \frac{{{\text{R}}_{\text{DAB}}^{{\prime \prime }} }}{{{\text{L}}_{\text{DAB}}^{{\prime \prime }} }}} & {\frac{1}{{{\text{L}}_{\text{DAB}}^{{\prime \prime }} }}} \\ { - \frac{1}{{{\text{C}}_{{{\text{DAB}}2}} }}} & { - \frac{1}{{{\text{R}}_{2} {\text{C}}_{{{\text{DAB}}2}} }}} \\ \end{array} } \right]\left[ {\begin{array}{*{20}c} {{\text{I}}_{{{\text{DAB}}2}} } \\ {{\text{U}}_{{{\text{DAB}}2}} } \\ \end{array} } \right] + \left[ {\begin{array}{*{20}c} {\frac{1}{{{\text{n}}_{\text{Tr}} {\text{L}}_{\text{DAB}}^{{\prime \prime }} }}} \\ 0 \\ \end{array} } \right]{\text{U}}_{\text{DAB1}}$$

36 $$\frac{\text{d}}{\text{dt}}{\text{x}} = {\text{A}}_{2} {\text{x}} + {\text{B}}_{2} {\text{u}} \Leftrightarrow \frac{\text{d}}{\text{dt}}\left[ {\begin{array}{*{20}c} {{\text{I}}_{{{\text{DAB}}2}} } \\ {{\text{U}}_{{{\text{DAB}}2}} } \\ \end{array} } \right] = \left[ {\begin{array}{*{20}c} { - \frac{{{\text{R}}_{\text{DAB}}^{{\prime \prime }} }}{{{\text{L}}_{\text{DAB}}^{{\prime \prime }} }}} & {\frac{1}{{{\text{L}}_{\text{DAB}}^{{\prime \prime }} }}} \\ { - \frac{1}{{{\text{C}}_{{{\text{DAB}}2}} }}} & { - \frac{1}{{{\text{R}}_{2} {\text{C}}_{{{\text{DAB}}2}} }}} \\ \end{array} } \right]\left[ {\begin{array}{*{20}c} {{\text{I}}_{{{\text{DAB}}2}} } \\ {{\text{U}}_{{{\text{DAB}}2}} } \\ \end{array} } \right] + \left[ {\begin{array}{*{20}c} {\frac{1}{{{\text{n}}_{\text{Tr}} {\text{L}}_{\text{DAB}}^{{\prime \prime }} }}} \\ 0 \\ \end{array} } \right]{\text{U}}_{\text{DAB1}}$$

where ${\text{L}}_{\text{DAB}}^{{\prime \prime }} = {\text{L}}_{\text{DAB}} /{\text{n}}_{\text{Tr}}^{2}$, ${\text{R}}_{\text{DAB}}^{{\prime \prime }} = {\text{R}}_{\text{DAB}} /{\text{n}}_{\text{Tr}}^{ 2}$. The small-signal model for DAB converter can be derived using Eqs. (35) and (36):

37 $${\text{s}}\left[ {\begin{array}{*{20}c} {\hat{\imath }_{{{\text{DAB}}2}} } \\ {{\hat{\text{u}}}{}_{{{\text{DAB}}2}}} \\ \end{array} } \right] = \left[ {\begin{array}{*{20}c} { - \frac{{{\text{R}}_{\text{DAB}}^{\prime \prime } }}{{{\text{L}}_{\text{DAB}}^{\prime \prime } }}} & {\frac{{2{\text{D}}_{\text{DAB}} - 1}}{{{\text{L}}_{\text{DAB}}^{\prime \prime } }}} \\ {\frac{{2{\text{D}}_{\text{DAB}} + 1}}{{{\text{C}}_{{{\text{DAB}}2}} }}} & { - \frac{1}{{{\text{R}}_{2} {\text{C}}_{{{\text{DAB}}2}} }}} \\ \end{array} } \right]\left[ {\begin{array}{*{20}c} {{\text{I}}_{{{\text{DAB}}2}} } \\ {{\hat{\text{u}}}_{{{\text{DAB}}2}} } \\ \end{array} } \right] + \left[ {\begin{array}{*{20}c} {\frac{1}{{{\text{n}}_{\text{Tr}} L_{\text{DAB}}^{\prime \prime } }}} \\ 0 \\ \end{array} } \right]{\hat{\text{u}}}_{{{\text{DAB}}1}} + \left[ {\begin{array}{*{20}c} {\frac{{2{\text{U}}_{{{\text{DAB}}2}} }}{{{\text{L}}_{\text{DAB}}^{\prime \prime } }}} \\ { - \frac{{2{\text{I}}_{{{\text{DAB}}2}} }}{{{\text{C}}_{{{\text{DAB}}2}} }}} \\ \end{array} } \right]{\hat{\text{d}}}_{\text{DAB}}$$

Small-signal transfer functions of DAB converter are control-to-output-current and output-current-to-DC voltage transfer functions. These transfer functions are as follow:

38 $${\text{G}}_{{{\text{DAB}}1}} = \frac{{\hat{\imath }_{{{\text{DAB}}2}} }}{{{\hat{\text{d}}}_{\text{DAB}} }} = \frac{{2{\text{U}}_{{{\text{DAB}}2}} }}{{{\text{sL}}_{{{\text{DAB}}2}}^{{\prime \prime }} + {\text{R}}_{\text{DAB}}^{{\prime \prime }} }}$$

39 $${\text{G}}_{{{\text{DAB}}2}} = \frac{{{\hat{\text{u}}}_{{{\text{DAB}}2}} }}{{\hat{\imath }_{\text{DAB}} }} = \frac{{{\text{R}}_{2} ( - 2{\text{D}}_{\text{DAB}} + 1)}}{{{\text{sR}}_{2} {\text{C}}_{{{\text{DAB}}2}} + 1}}$$

**Fig. 12:**
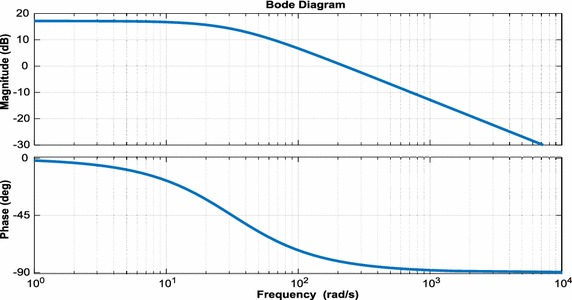
Bode plots of phase shift to output voltage for small signal model

Moreover, bode plots of the simplified linearized model are given in Fig. 12.

### Control of the output stage

**Fig. 13:**
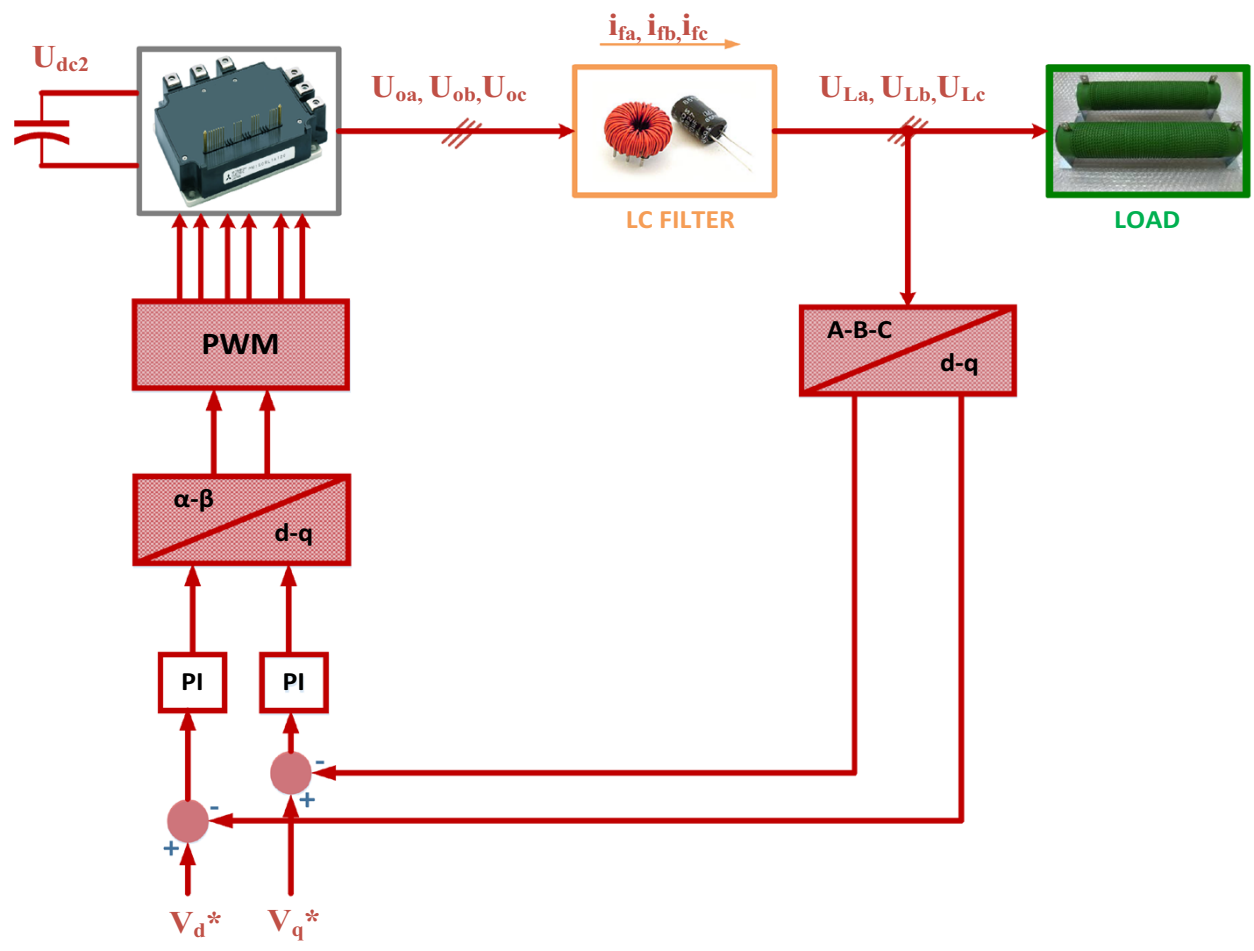
Configuration of three-phase two-level inverter

Output stage consists of three-phase inverter, LC filter and load as seen in Fig. 13. Three-phase inverter used in the output stage has six directional switches which converts DC voltage into three-phase AC voltages. We proposed PWM technique for three-phase inverter. In the output stage, three-phase output voltages are first converted to voltages of d–q axis (V_d_, V_q_) in the synchronous rotating d-q reference frame. Then, these voltages are compared with the reference values of V_d_ and V_q_. The outputs of PI controller are transformed to U_α_ − U_β_ voltage which is used to generate inverter gate pulses (Liu et al. 2009; Hooshmand et al. 2012). Equations of these transformations are given as follows:

40 $$\left[ {\begin{array}{*{20}c} {{\text{U}}_{\text{oa}} } \\ {{\text{U}}_{\text{ob}} } \\ {{\text{U}}_{\text{oc}} } \\ \end{array} } \right] = {\text{L}}\frac{\text{d}}{\text{dt}}\left[ {\begin{array}{*{20}c} {{\text{I}}_{\text{fa}} } \\ {{\text{I}}_{\text{fb}} } \\ {{\text{I}}_{\text{fc}} } \\ \end{array} } \right] + \left[ {\begin{array}{*{20}c} {{\text{U}}_{\text{La}} } \\ {{\text{U}}_{\text{Lb}} } \\ {{\text{U}}_{\text{Lc}} } \\ \end{array} } \right]$$

41 $$\left[ {\begin{array}{*{20}c} {{\text{U}}_{\text{od}} } \\ {{\text{U}}_{\text{oq}} } \\ \end{array} } \right] = {\text{L}}\frac{\text{d}}{\text{dt}}\left[ {\begin{array}{*{20}c} {{\text{I}}_{\text{d}} } \\ {{\text{I}}_{\text{q}} } \\ \end{array} } \right] + \left[ {\begin{array}{*{20}c} {{\text{U}}_{\text{Ld}} } \\ {{\text{U}}_{\text{Lq}} } \\ \end{array} } \right] + \omega {\text{L}}\left[ {\begin{array}{*{20}c} { - {\text{I}}_{\text{q}} } \\ {{\text{I}}_{\text{d}} } \\ \end{array} } \right]$$

where, d_d_ and d_q_ are duty cycles corresponding to the dq-axes respectively. Equations (40) and (41) are obtained (Hiti et al. 1994; Tuomas et al. 2015);

42 $${\text{d}}_{\text{d}} \cdot {\text{U}}_{{{\text{dc}}2}} = {\text{L}}\frac{{{\text{di}}_{\text{d}} }}{\text{dt}} - \omega {\text{Li}}_{\text{q}} + {\text{U}}_{\text{Ld}}$$

43 $${\text{d}}_{\text{q}} \cdot {\text{U}}_{{{\text{dc}}2}} = {\text{L}}\frac{{{\text{di}}_{\text{q}} }}{\text{dt}} - \omega {\text{Li}}_{\text{d}} + {\text{U}}_{\text{Lq}}$$

Moreover, an operating point is defined to conduct small-signal model and to analyze the three-phase two-level inverter used in EPT structure. For small signal model, a perturbation is given around the operating point which is given as follows:

44 $${\text{U}}_{{{\text{dc}}2}} = {\text{U}}_{{{\text{dc}}2}} + {\hat{\text{U}}}_{\text{dc2}}$$

45 $${\text{D}}_{\text{d}} = {\text{D}}_{\text{d}} + {\hat{\text{d}}}_{\text{d}}$$

46 $${\text{D}}_{\text{q}} = {\text{D}}_{\text{q}} + {\hat{\text{d}}}_{\text{d}}$$

47 $${\text{i}}_{\text{d}} = {\text{i}}_{\text{d}} + {\hat{\text{i}}}_{\text{d}}$$

48 $${\text{i}}_{\text{q}} = {\text{i}}_{\text{q}} + {\hat{\text{i}}}_{\text{q}}$$

49 $${\text{U}}_{\text{Ld}} = {\text{U}}_{\text{Ld}} + {\hat{\text{U}}}_{\text{Ld}}$$

50 $${\text{U}}_{\text{Lq}} = {\text{U}}_{\text{Lq}} + {\hat{\text{U}}}_{\text{Lq}}$$

51 $${\hat{\text{i}}} = {\hat{\text{D}}}_{\text{d}} {\hat{\text{i}}}_{\text{d}} + {\hat{\text{D}}}_{\text{d}} {\hat{\text{i}}}_{\text{d}} + {\hat{\text{d}}}_{\text{d}} {\hat{\text{i}}}_{\text{d}} + {\hat{\text{d}}}_{\text{d}} {\hat{\text{i}}}_{\text{d}}$$

**Fig. 14:**
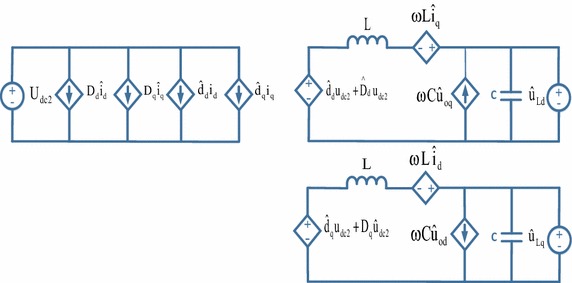
Small signal model of three-phase inverter

In the above new operating point, parameters with “^” are small perturbed variables. U_dc2_ is DC voltage of three-phase inverter, D_d_ is d-axis duty cycle, D_q_ is q-axis duty cycle, i_d_ is d-axis current, i_q_ is q-axis current, V_Ld_ and V_Lq_ are dq-axis grid voltages (Hiti et al. 1994; Tuomas et al. 2015). Based on the above equations, small signal model of three-phase inverter has been derived and shown in Fig. 14.

**Fig. 15:**
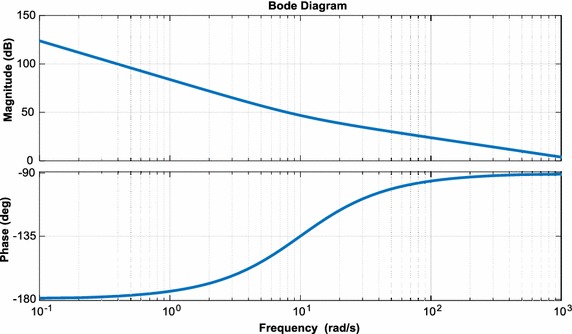
Bode plots of control loop for three-phase inverter

According to transfer function obtained from small signal model, bode plots of control loop for three-phase inverter are shown in Fig. 15. The bandwidth of the control loop is made low so that the DC link voltage control will not cause disturbances in the sinusoidal output currents of the three-phase inverter. The bandwidth of the control loop is adjusted near 100 Hz and the control system has a low steady state error.

## Simulation results

In this section, EPT structure is implemented in MATLAB/Simulink environment and the results of simulation studies are carried out in order to demonstrate dynamic performance of EPT structure under voltage harmonics, voltage flicker, voltage sag and swell conditions. Moreover, the parameters of EPT structure used in the simulation studies are compiled in Table 3.

**Table 3:** Parameters of EPT structure used in simulation study

Parameters	Value
Grid voltage	800 V_rms_
DC voltage of input stage	2000 V
DC voltage of isolation stage	400 V
Power frequency	50 Hz
Grid resistance and inductance	0.1 Ω, 5 mH
HF transformer	5:1, 1000 Hz, 30 kVA
Capacitors	3.3 mF, 4.7 mF
LC filter	2 mH, 200 µF

**Fig. 16:**
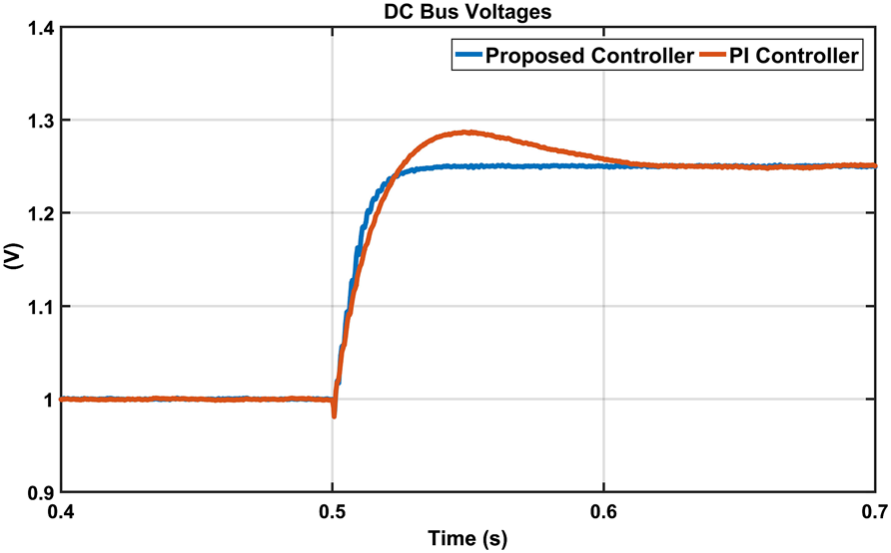
DC bus voltage responses of both controllers

DC bus voltage responses of the proposed controller and PI controller are shown in Fig. 16. DC voltage is adjusted to per-unit (p.u) reference value of 1. Then p.u reference value is increased to 1.25 p.u at t = 0.5 s. It can be seen from Fig. 16 that the proposed controller reaches to reference DC voltage after nearly 0.025 s without overshoot while PI controller reaches to reference DC voltage after 0.1 s with overshoot. Figure 16 shows that the proposed controller has superior properties compared with PI controller in terms of rise time, settling time and overshoot.

**Fig. 17:**
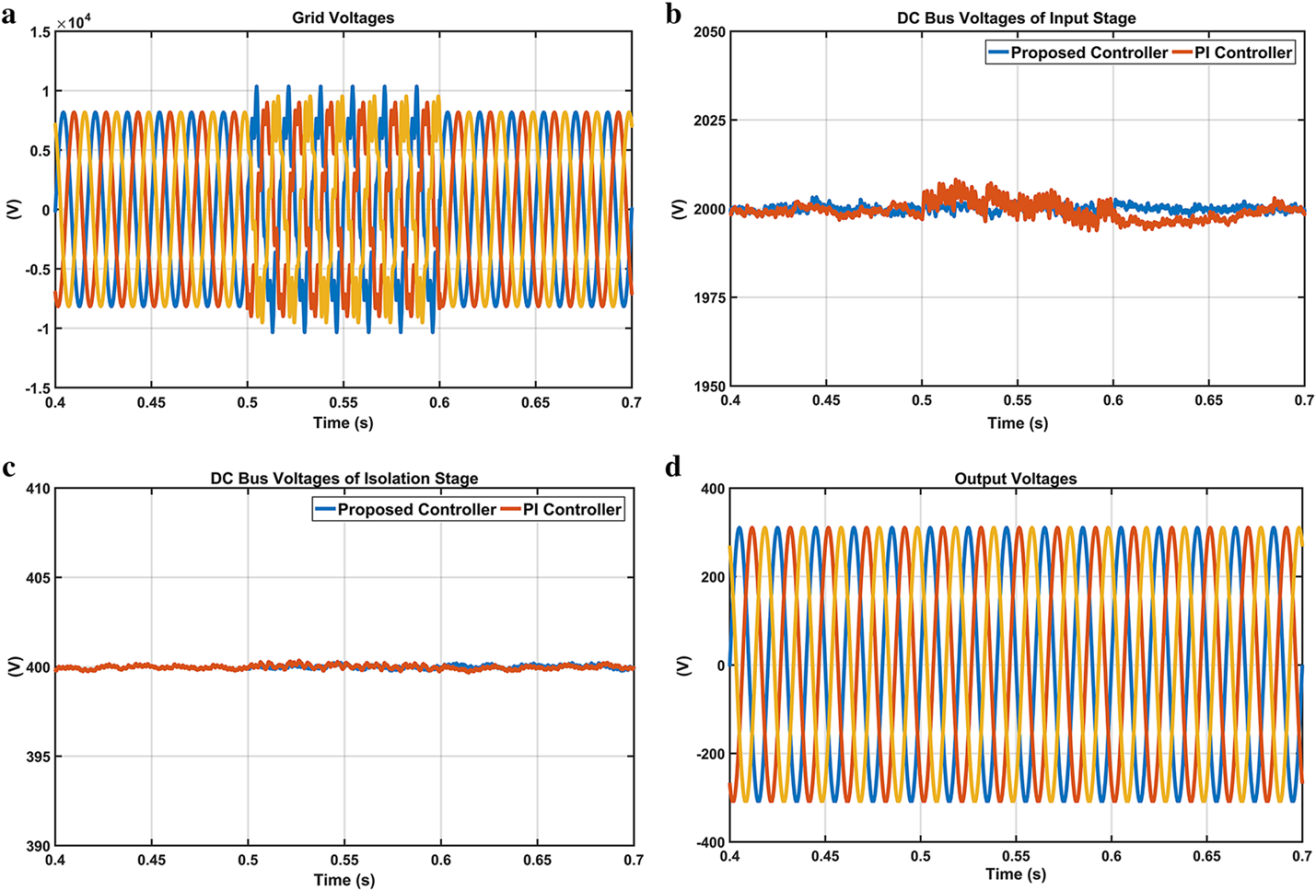
Waveforms of EPT structure under voltage harmonics condition. **a** Grid voltages, **b** DC bus voltage responses of input stage, **c** DC bus voltage responses of isolation stage and **d** output voltages

The first scenario is realized in order to indicate effectiveness of both controllers against voltage harmonics and waveforms of this situation are given in Fig. 17. Voltage harmonics of the 5th and 7th orders with amplitudes of 10 and 15 % are applied on the grid voltages between t = 0.5 and t = 0.6 s. When voltage harmonics condition occurs at t = 0.5 s, PI controller has oscillations while the proposed controller is not affected by this situation as seen Fig. 17b, c which show DC voltage responses of the isolation stage with DAB converter. Thanks to DAB converter with the proposed controller, the effect of the voltage harmonics is eliminated in the input stage. It has been observed from these waveforms that the proposed controller have better performance than PI controller with regard to settling time, oscillation and overshoot. Moreover, Fig. 17d shows clearly that the output voltages are not affected by voltage harmonics in grid.

**Fig. 18:**
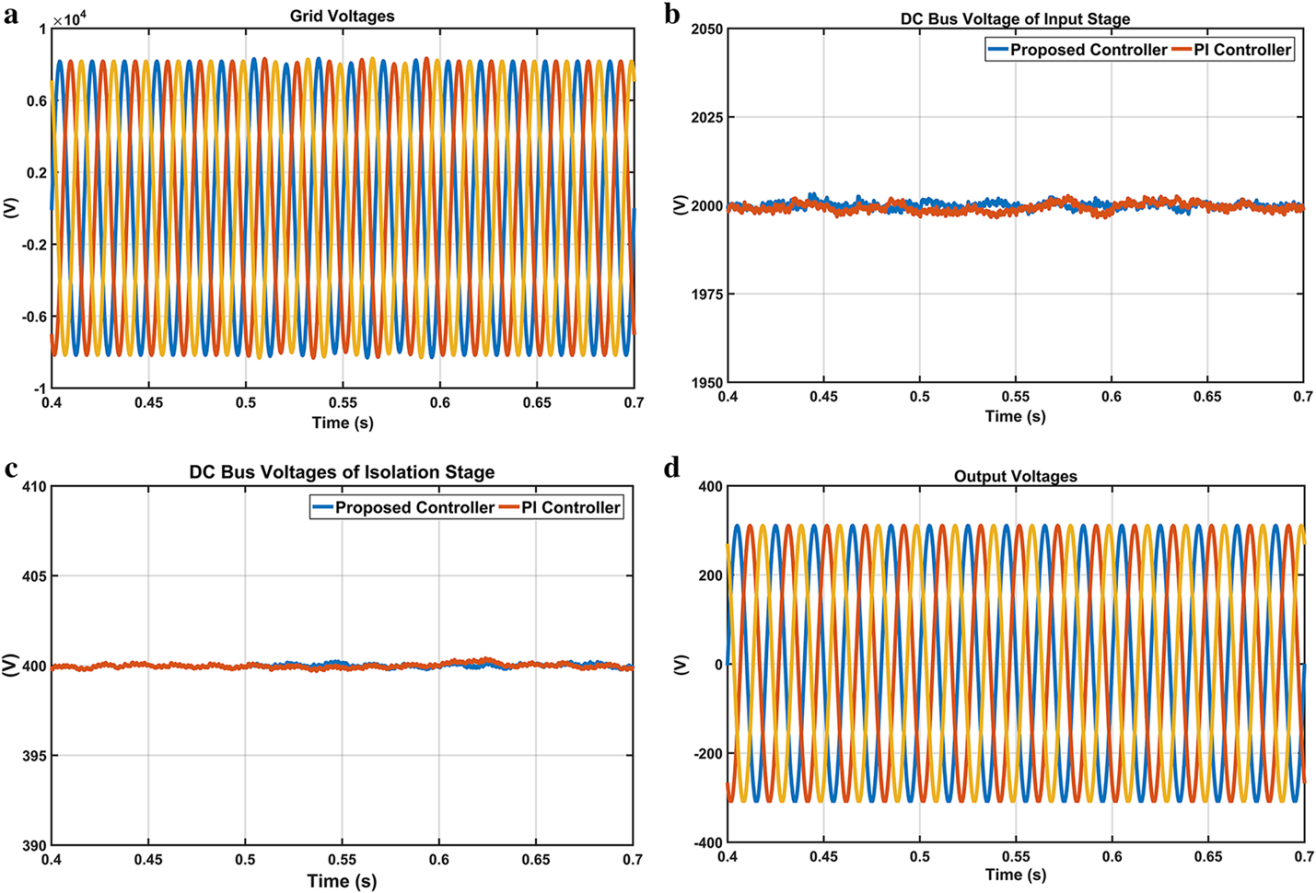
Waveforms of EPT structure under voltage flicker case. **a** Grid voltages, **b** DC bus voltage responses of input stage, **c** DC bus voltage responses of isolation stage and **d** output voltages

The second scenario is performed in order to demonstrate performance of both controllers under a voltage flicker and waveforms of this condition are given in Fig. 18. The grid voltages are first set to sinusoidal wave shape with a frequency of 15 Hz and a modulation index of 10 %. Then this flicker condition is formed between t = 0.5 and 0.6 s. As shown in Fig. 18b, DC voltage responses obtained from both controllers have a small oscillation. DC voltage responses of the isolation stage based on DAB converter are shown in Fig. 18c. It is observed that isolation stage is not affected by flicker condition as shown in Fig. 18c. Besides, this flicker case is removed in the output voltages and consequently, regulated output voltages are obtained.

**Fig. 19:**
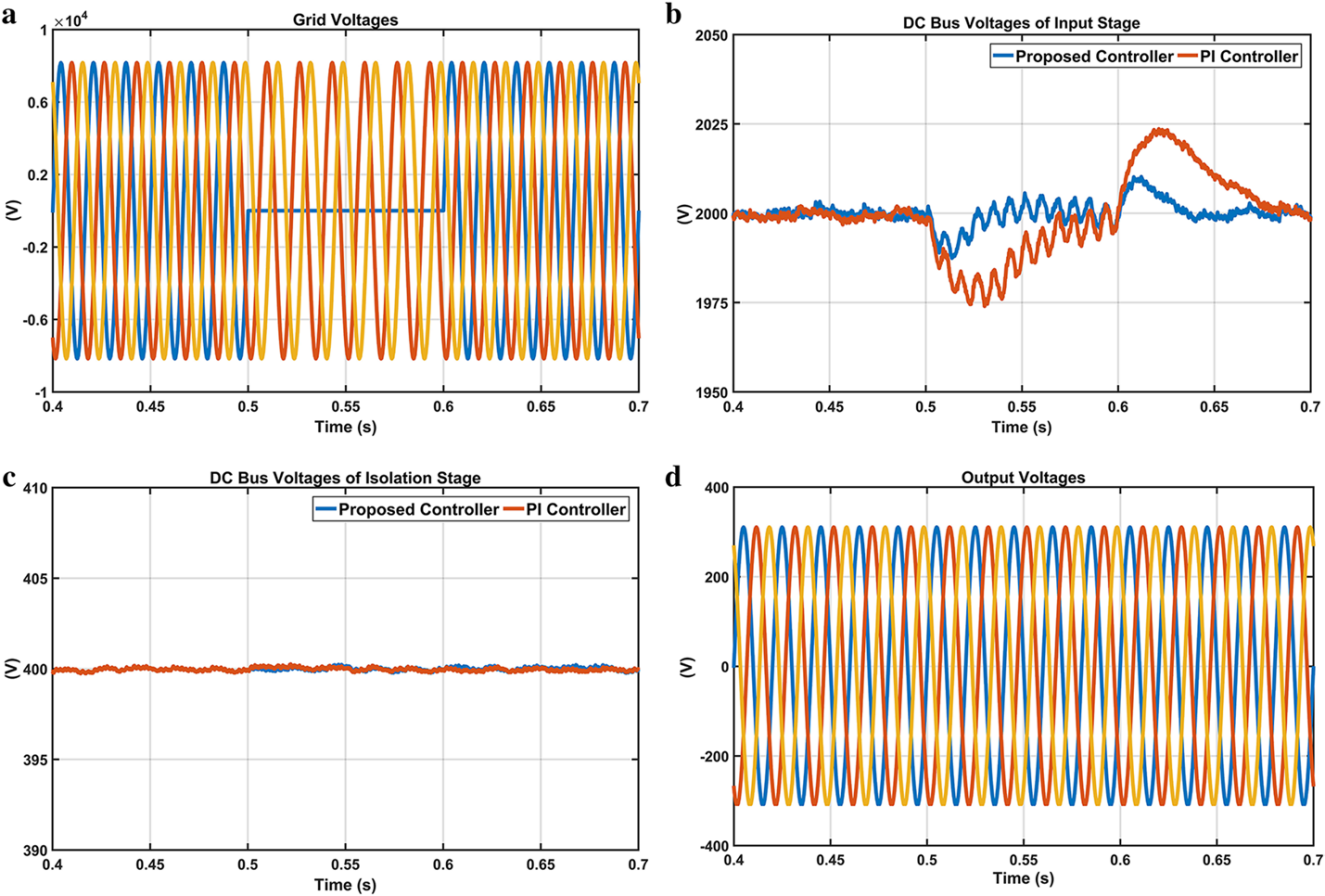
Waveforms of EPT structure under voltage flicker case. **a** Grid voltages, **b** DC bus voltage responses of input stage, **c** DC bus voltage responses of isolation stage and **d** output voltages

The third scenario is carried out in order to verify the performance of the proposed controller and PI controller during 100 % voltage sag condition in phase A. Figure 19 shows waveforms when there is 100 % of phase-A voltage sag from t = 0.5–0.6 s. According to enlarged DC voltage responses given in Fig. 19b, when the voltage sag occurs in phase-A, DC voltage response obtained from PI controller falls 1975 V and reaches reference DC voltage at 0.7 s while the proposed controller falls 1990 V and rapidly reaches reference DC voltage at 0.63 s. Waveforms related to the isolation stage given in Fig. 19c are illustrated that the proposed controller has more efficient performance than PI controller in this condition. Also, the output voltages have clearly sinusoidal and symmetrical forms as it is seen from Fig. 19d.

**Fig. 20:**
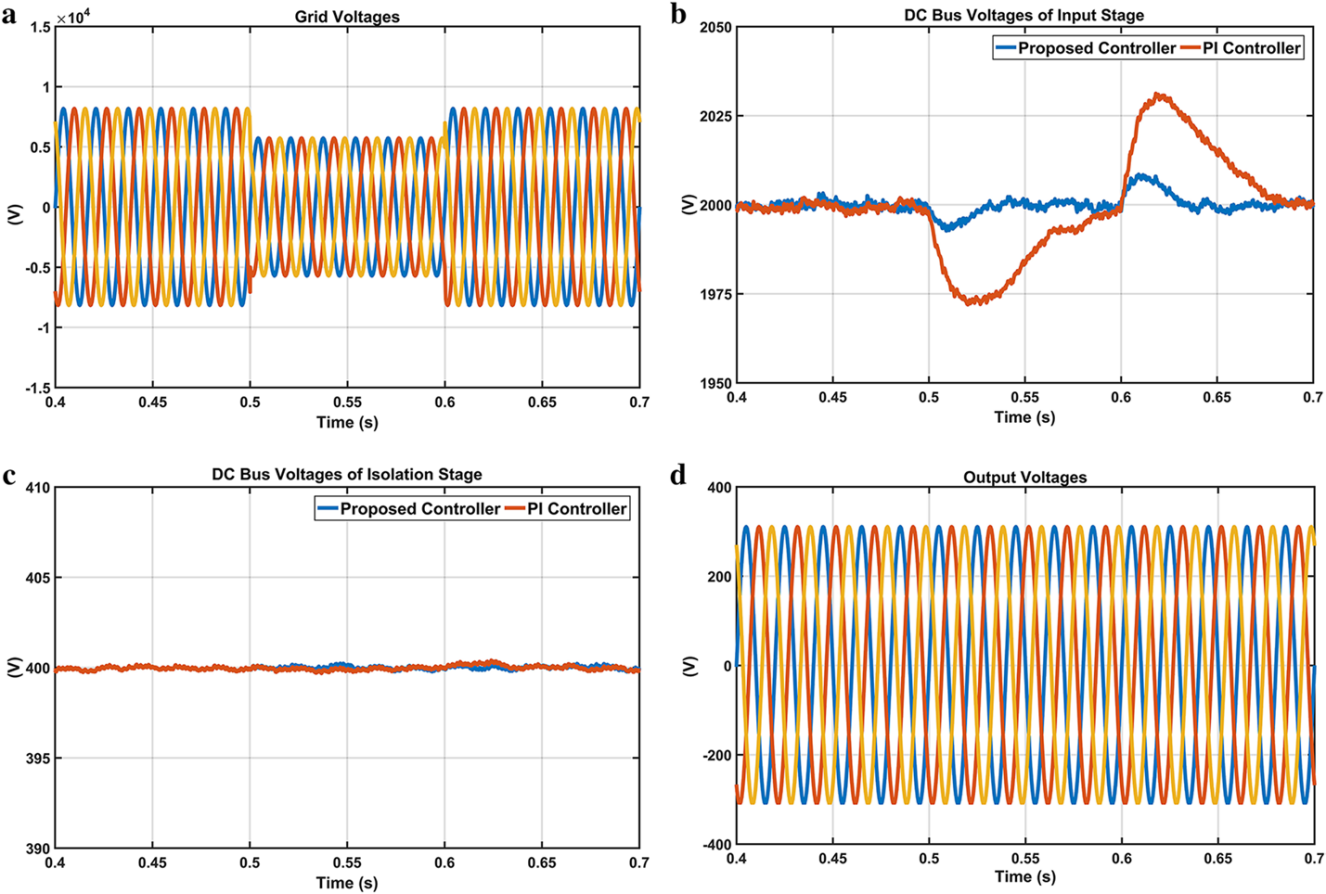
Waveforms of EPT structure under voltage sag condition. **a** Grid voltages, **b** DC bus voltage responses of input stage, **c** DC bus voltage responses of isolation stage and **d** output voltages

The fourth scenario is carried out to demonstrate response of both controllers under voltage sag condition as shown in Fig. 20. In this scenario, the magnitude of grid voltage changes from 100 to 70 % at 0.5 s, and then changes back from 70 to 100 % at 0.6 s. When the voltage sag happens at t = 0.5 s, enlarged DC voltage obtained from PI controller falls nearly 1974 V and reaches reference DC voltage at 0.7 s whereas the proposed controller falls 1991 V and reaches reference DC voltage at 0.63 s. As clearly seen in Fig. 20c, although voltage sag at the input stage is occurred from 0.5 to 0.6 s, DC response obtained from the proposed controller is more durable than PI controller. Moreover, Fig. 20d shows that the output voltages are remained fixed and sinusoidal in spite of voltage sag conditions.

**Fig. 21:**
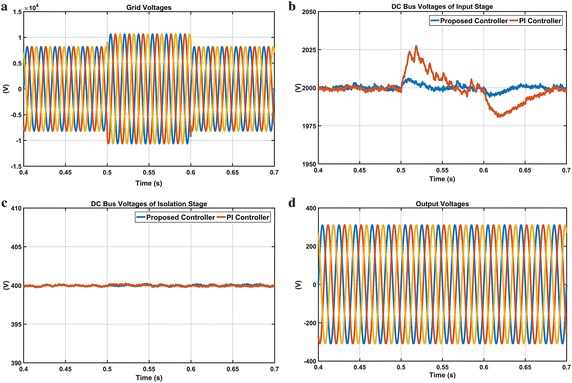
Waveforms of EPT structure under voltage swell condition. **a** Grid voltages, **b** DC bus voltage responses of input stage, **c** DC bus voltage responses of isolation stage and **d** output voltages

The last scenario is realized in order to demonstrate the performance of both controllers during voltage swell condition. Figure 21 shows waveforms when the grid voltages occur 30 % of three-phase voltage swells at t = 0.5 to 0.6 s. According to Fig. 21, when the voltage swell happens, the proposed controller rises 2004 V and reaches reference DC voltage at 0.63 s whereas DC voltage response obtained from PI controller rises 2028 V and reaches reference DC voltage at 0.7 s. Figure 21c shows waveforms of the isolation stage in this scenario. Isolation stage based on DAB converter with the proposed controller is more successful than PI controller in eliminating effect of the voltage swell in the input stage and the proposed controller has much better reference tracking without steady-state error after voltage swell occurs. Besides, Fig. 21d clearly indicates that proposed EPT is able to regulate output voltages and compensate voltage swell in grid voltages.

## Conclusion

EPT structure which has many superior features such as high power factor, voltage sag/swell compensation, multi-functionality, excellent power quality compared with conventional transformer is proposed in this study. EPT structure in this study is composed of input, isolation and output stages. Three-phase PWM rectifier at the input stage is not only used in order to convert AC to the constant DC voltage, but also has reactive power compensation ability. PI controllers are generally used in PWM rectifiers due to their simple structures. However, PI controller needs mathematical model of the system to be controlled and has undesirable characteristics such as slow response, large overshoots and oscillation. To cope with these problems, neuro-fuzzy controller that has nonlinear, robust structure and which does not require the mathematical model of the system to be controlled is preferred in this study. Dual active bridge converter at isolation stage is used for DC–DC conversion and is controlled by neuro-fuzzy controller in order to obtain constant DC bus voltage. Three-phase inverter that provides the desired power and voltage to load is located at the output stage. After designing of all stages, a number of simulation studies have been carried out in order to verify performance of EPT structure with the proposed controller under voltage harmonics, voltage flicker and voltage sag/swell conditions. The simulation results illustrate that EPT structure with the neuro-fuzzy controller provides more superior performance than PI controller with respect to rise time, settling time, overshoot, and power factor in all test conditions and is not sensitive these conditions and is capable of regulating output voltages and compensating disturbances in grid voltages. Moreover, proposed EPT provides fast and controllable AC/DC responses because of strong structure of the neuro fuzzy controller and thus, improves the stability of power system.
